# Gene organization and sequence analyses of transfer RNA genes in Trypanosomatid parasites

**DOI:** 10.1186/1471-2164-10-232

**Published:** 2009-05-18

**Authors:** Norma E Padilla-Mejía, Luis E Florencio-Martínez, Elisa E Figueroa-Angulo, Rebeca G Manning-Cela, Rosaura Hernández-Rivas, Peter J Myler, Santiago Martínez-Calvillo

**Affiliations:** 1 Unidad de Biomedicina, Facultad de Estudios Superiores Iztacala, Universidad Nacional Autónoma de México, Av de los Barrios 1, Col Los Reyes Iztacala, Tlalnepantla, Edo de México, CP 54090, México; 2 Departamento de Biomedicina Molecular, Centro de Investigación y de Estudios Avanzados del IPN, Apartado Postal 14-740, 07360, México, DF, México; 3 Seattle Biomedical Research Institute, 307 Westlake Avenue N, Seattle, WA 98109-5219, USA; 4 Departments of Global Health, and Medical Education & Biomedical Informatics, University of Washington, Seattle, WA 98195, USA

## Abstract

**Background:**

The protozoan pathogens *Leishmania major*, *Trypanosoma brucei *and *Trypanosoma cruzi *(the Tritryps) are parasites that produce devastating human diseases. These organisms show very unusual mechanisms of gene expression, such as polycistronic transcription. We are interested in the study of tRNA genes, which are transcribed by RNA polymerase III (Pol III). To analyze the sequences and genomic organization of tRNA genes and other Pol III-transcribed genes, we have performed an *in silico *analysis of the Tritryps genome sequences.

**Results:**

Our analysis indicated the presence of 83, 66 and 120 genes in *L. major, T. brucei *and *T. cruzi*, respectively. These numbers include several previously unannotated selenocysteine (Sec) tRNA genes. Most tRNA genes are organized into clusters of 2 to 10 genes that may contain other Pol III-transcribed genes. The distribution of genes in the *L. major *genome does not seem to be totally random, like in most organisms. While the majority of the tRNA clusters do not show synteny (conservation of gene order) between the Tritryps, a cluster of 13 Pol III genes that is highly syntenic was identified. We have determined consensus sequences for the putative promoter regions (Boxes A and B) of the Tritryps tRNA genes, and specific changes were found in tRNA-Sec genes. Analysis of transcription termination signals of the tRNAs (clusters of Ts) showed differences between *T. cruzi *and the other two species. We have also identified several tRNA isodecoder genes (having the same anticodon, but different sequences elsewhere in the tRNA body) in the Tritryps.

**Conclusion:**

A low number of tRNA genes is present in Tritryps. The overall weak synteny that they show indicates a reduced importance of genome location of Pol III genes compared to protein-coding genes. The fact that some of the differences between isodecoder genes occur in the internal promoter elements suggests that differential control of the expression of some isoacceptor tRNA genes in Tritryps is possible. The special characteristics found in Boxes A and B from tRNA-Sec genes from Tritryps indicate that the mechanisms that regulate their transcription might be different from those of other tRNA genes.

## Background

The parasites *Leishmania major*, *Trypanosoma brucei *and *Trypanosoma cruzi*, referred together as Tritryps, are trypanosomatid protozoa that cause deadly human diseases known as leishmaniasis, African sleeping sickness and Chagas disease, respectively. Collectively, these pathogens cause millions of deaths in developing countries in tropical and subtropical regions of the world. Analyses of the recently reported genomic sequences of the Tritryps revealed a striking feature: their genomes are organized into large directional gene clusters, *i.e. *tens-to-hundreds of protein-coding genes arranged sequentially on the same strand of DNA [1-3]. Transcription of the gene clusters is polycistronic, and mature mRNAs are generated from long precursors by trans-splicing and polyadenylation [4,5]. Most chromosomes contain at least two polycistronic gene clusters (PGCs), which can be either divergently transcribed (towards the telomeres) or convergently transcribed (away from the telomeres). Chromosome 3 from *L. major *contains two convergent PGCs (of 67 and 45 genes) that are separated by a tRNA gene. Interestingly, Pol II-transcription of both PGCs terminates within the tRNA-gene region [6]. The *L. major *nuclear genome is distributed among 36 relatively small chromosomes that range from 0.28 to 2.8 Mb. *T. cruzi *possesses ~28 medium-sized chromosomes, while *T. brucei *has 11 large chromosomes. Regardless of having diverged more than 200 million years ago, the genomes of trypanosomatids show a remarkable conservation of gene order (synteny) [7].

We are interested in the study of transcription by RNA polymerase III (Pol III), which produces small essential RNA molecules, such as tRNA [8]. All tRNAs have sequences of 74–95 bases that fold into a characteristic cloverleaf secondary structure with four constant arms. The acceptor arm binds to a particular amino acid, specified by the anticodon triplet located in the anticodon arm. The anticodon is complementary to an mRNA codon, specific for the amino acid carried by the tRNA. Therefore, tRNAs serve as adaptor molecules that mediate the transfer of information from nucleic acid to protein [9]. Organisms must have at least one tRNA for each of the 20 amino acids. Because different types of relaxed base pairings are allowed at the "wobble" position of the anticodon, certain tRNAs (known as isoacceptors) can read two or more synonymous codons differing by the third base. Consequently, cells do not carry tRNAs with anticodons complementary to all of the 61 possible codons in the genetic code. Interestingly, several organisms contain a large proportion of tRNA genes that have the same anticodon sequence, but differences elsewhere in the tRNA body [10]. The number of these tRNA genes, called isodecoders, varies from very low (10 in yeast) to very high (225–246) in chimp and human. Thus, the diversity of tRNA genes is higher than originally thought [10].

Most organisms usually contain several hundred tRNA genes distributed randomly over their entire genome. One of the distinctive features of most genes transcribed by Pol III is that their promoter sequences are located within the transcribed region. In the case of tRNA genes, the promoter consists of two conserved elements: Boxes A and B. While Box A is normally positioned close to the transcription start site, the location of Box B is variable, partly because some tRNAs have short introns within the coding region [8,11].

Here we report the *in silico *analysis of tRNA genes in trypanosomatids. We found that, unlike in most other organisms, the distribution of genes in the genomes of *L. major *and *T. brucei *does not seem to be totally random, being confined to a subset of chromosomes. In addition, 14 out of 39 convergent strand-switch regions found in *L. major *contain at least one tRNA gene, which suggests that the use of tRNA genes as signals for termination of transcription of PGCs might be a common process in this parasite. Our analysis also indicated that the majority of the tRNA clusters do not show conservation of gene order among Tritryps. Analysis of the putative transcription termination signals in all the tRNA genes showed an average of 5 Ts (+/- 1) in *L. major *and *T. brucei*, and 6 Ts (+/- 2) in *T. cruzi*. Also, special features were found in promoter elements from tRNA-Sec genes from Tritryps. Finally, we have identified several tRNA isodecoder genes in the Tritryps.

## Results and discussion

### Number of tRNA genes

**Figure 1:**
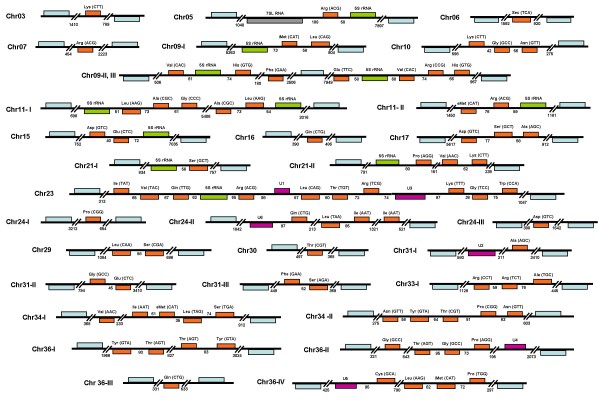
**Organization of tRNA genes in *L. major***. The 83 tRNA genes found in the genome of the parasite are shown in orange. The predicted anticodons are indicated in parentheses. 5S rRNA, snRNA and 7SL genes are shown in green, purple and gray, respectively. Genes are drawn to scale, and the sizes of intergenic regions are indicated (in base pairs). Protein-coding genes that flank Pol III-transcribed genes are shown in blue (not to scale). The tRNA-Sec gene on chromosome 6 is located at positions 69,586 to 69,673, in the complement strand. Putative pseudogenes are not shown. For practical purposes, we regarded protein-coding genes as the limits of a particular Pol III locus. For that reason, we considered cluster chr09-II, III as two independent Pol III loci. More of such cases are shown in Fig. 2.

**Figure 2:**
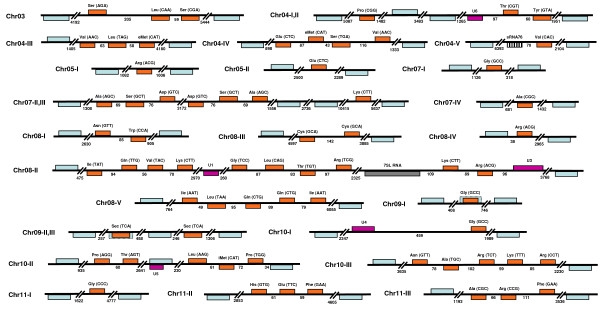
**Organization of tRNA genes in *T. brucei***. The 66 tRNA genes, distributed into 23 loci, are indicated in orange. The predicted anticodons are shown in parentheses. snRNA and 7SL genes are shown in purple and gray, respectively. The sRNA76 (misannotated in GeneDB as a tRNA-Sec gene) is shown as a stripped box in locus chr04-V. The two tRNA-Sec genes that we found are located in loci chr09-II, III (at positions 505,606 to 505,693 and 509,443 to 509,530 of chromosome 9). The gene from locus chr09-II overlaps a putative Pol II gene (Tb09.160.1080, dotted blue box), annotated as hypothetical protein (unlikely). Also, the tRNA-Gly from locus chr09-I overlaps a putative Pol II gene (Tb09.211.4080, dotted blue box), annotated as hypothetical protein (unlikely). The U5 snRNA gene (locus chr10-II) also overlaps a Pol II gene (Tb10.6k15.2990, sequence orphan), but located on the opposite strand. Genes are drawn to scale, and the sizes of intergenic regions are indicated (in base pairs). Protein-coding genes that flank Pol III-transcribed genes are shown in blue (not to scale). In locus chr08-V, the Leu-TAA gene (Tb_08_TRNA_Leu_2) and the first Gln-CTG gene (Tb_08_TRNA_Gln_2) are annotated in the wrong (opposite) strand in GeneDB. Maps of an incomplete repertoire of tRNA genes from *T. brucei *were previously reported [16].

Analysis of the GeneDB databases from *L. major*, *T. brucei *and *T. cruzi *(Tritryps) revealed the presence of 82, 65 and 115 tRNA genes, respectively (see Table 1 and Additional File 1). By using the tRNAscan-SE program, we confirmed the identity of all the annotated tRNA genes in *L. major *(Fig. 1). However, we found a few discrepancies in the *T. brucei *and *T. cruzi *annotated genomes. In the case of *T. brucei*, it was observed that the tRNA-Sec (Tb04_tRNA-SeC1) gene annotated on chromosome 4 actually corresponds to the sRNA76 (see cluster chr04-V in Fig. 2), which is a tRNA-like molecule that associates to the 7SL RNA in trypanosomatids [12]. By doing a search of the *T. brucei *genome with the tRNA-Sec gene sequence reported previously [13], we located two copies of the genuine gene on chromosome 9 (see clusters chr09-II-III in Fig. 2). One of these tRNA genes is located within an open reading frame (ORF), annotated as a "hypothetical protein, unlikely" (Tb09.160.1080). We also identified one tRNA-Sec gene in *L. major *(see cluster chr06 in Fig. 1). Interestingly, eight tRNA-Sec genes were found in the *T. cruzi *genome (see Additional File 1); they all are organized as independent genes, not clustered with other Pol III genes (data not shown). The tRNA-Sec is a component of a translational mechanism that reads UGA (normally a stop codon) as a selenocysteine codon in selected mRNAs that contain a specific *cis*-acting RNA regulatory sequence in their 3' untranslated regions (3'-UTRs) [14]. The presence of selenoproteins, and all the machinery required for its synthesis, has been demonstrated in *L. major *and *T. cruzi *[13,15].

**Table 1:** Repertoire of tRNA genes and codon usage in Tritryps.

**Amino acid**	Codon	tDNA anticodon	*L. major *gene copy number	*T. brucei *gene copy number	*T. cruzi *gene copy number	Anticodon^2^	*L. major *Codon usage %	*T. brucei *Codon usage %	*T. cruzi *Codon usage %
**Ala**	GCT	AGC	2	2	2 (98%) ^1^	**IGC**	1.83	2.08	1.61
	GCC	GGC	0	0	0		3.61	1.82	1.62
	GCA	TGC	1	1	2 (97%)^1^	UGC	2.02	2.32	2.16
	GCG	CGC	2	2 (97%) ^1^	2	CGC	3.62	2.07	2.36

**Arg**	CGT	ACG	4	3	4 (98%) ^1^	**ICG**	1.05	1.59	1.60
	CGC	GCG	0	0	0		3.18	1.45	1.75
	CGA	TCG	1	1	2	UCG	0.73	0.9	1.58
	CGG	CCG	1	1	2	CCG	1.38	1.21	1.85
	AGA	TCT	1	1	2	UCU	0.27	0.68	1.56
	AGG	CCT	1	1	2	CCU	0.55	1	1.86

**Asn**	AAT	ATT	0	0	0	**GUU**	0.53	1.82	1.37
	AAC	GTT	3	2	4		2.07	1.92	1.45

**Asp**	GAC	GTC	3	2	2	**GUC**	3.42	2.27	1.60
	GAT	ATC	0	0	0		1.44	2.81	1.57

**Cys**	TGT	ACA	0	0	0	**GCA**	0.42	1.09	1.78
	TGC	GCA	1	2	2		1.46	1.13	2.34

**Gln**	CAA	TTG	1	1	2	UUG	0.76	1.69	1.66
	CAG	CTG	3	2	4	CUG	3.32	2.1	1.8

**Glu**	GAA	TTC	1	1	1	UUC	1.14	3.17	2.17
	GAG	CTC	2	2	4 (95%) ^1^	CUC	3.32	3.81	2.21

**Gly**	GGT	ACC	0	0	0	**GCC**	1.25	2.27	1.51
	GGC	GCC	4	3	4		3.36	1.49	1.95
	GGA	TCC	1	1	2	UCC	0.64	1.56	2.16
	GGG	CCC	1	1	2	CCC	1.19	1.39	1.64

**His**	CAT	ATG	0	0	0	**GUG**	0.65	1.13	1.37
	CAC	GTG	2	1	4 (98%) ^1^		2.04	1.3	1.64

**Ile**	ATT	AAT	3	2	4	**IAU**	0.82	1.9	1.42
	ATC	GAT	0	0	0		1.88	1.16	1.10
	ATA	TAT	1	1	2	UAU	0.27	1	0.75

**Leu**	TTA	TAA	1	1	2	UAA	0.16	0.98	0.89
	TTG	CAA	1	1	2	CAA	1.1	1.96	2.33
	CTT	AAG	3	1	4	**IAG**	1.11	2.23	1.59
	CTC	GAG	0	0	0		2.5	1.56	1.27
	CTA	TAG	1	1	2	UAG	0.47	0.82	0.54
	CTG	CAG	2	1	2	CAG	3.83	1.85	1.95

**Lys**	AAA	TTT	1	1	2	UUU	0.54	2.06	1.94
	AAG	CTT	3	3	4	CUU	2.78	2.66	1.88

**Met**	ATG	CAT	4	3	6	CAU	2.25	2.34	2.12

**Phe**	TTT	AAA	0	0	0	**GAA**	1.04	2.05	2.17
	TTC	GAA	2	2	4		1.91	1.59	1.49

**Pro**	CCT	AGG	2	1	2	**IGG**	0.86	1.11	1.05
	CCC	GGG	0	0	0		1.24	1.11	1.03
	CCA	TGG	1	1	2	UGG	1.05	1.39	1.44
	CCG	CGG	2	1	2	CGG	2.61	1.18	1.57

**Ser**	TCT	AGA	1	1	2	**IGA**	1.02	1.26	1.37
	TCC	GGA	0	0	0		1.69	1.25	1.24
	TCA	TGA	1	1	2 (98%) ^1^	UGA	0.73	1.35	1.41
	TCG	CGA	1	1	2	CGA	2.17	1.16	1.2
	AGT	ACT	0	0	0	**GCU**	0.73	1.51	1.21
	AGC	GCT	2 (98%) ^1^	2	2		2.6	1.35	1.60

**Thr**	ACT	AGT	3	1	2	**IGU**	0.68	1.3	1.13
	ACC	GGT	0	0	0		1.77	1.21	1.18
	ACA	TGT	1	1	2	UGU	1.04	1.74	1.74
	ACG	CGT	2	1	2	CGU	2.48	1.48	1.98

**Trp**	TGG	CCA	1	1	2	CCA	1.07	1.09	2.33

**Tyr**	TAT	ATA	0	0	0	**GUA**	0.4	1.13	0.84
	TAC	GTA	3	1	2		1.99	1.41	1.04

**Val**	GTT	AAC	2	2	2	**IAC**	0.86	2.29	1.49
	GTC	GAC	0	0	0		1.92	1.14	1.26
	GTA	TAC	1	1	2	UAC	0.54	1.26	0.83
	GTG	CAC	2	1	1	CAC	3.82	2.87	2.65

**SeC**	TGA	TCA	1	2	8				
				
**TOTAL**			83	66	120				

^1 ^One isodecoder tRNA was identified in *L. major*, one in *T. brucei *and six in *T. cruzi*. The percentage of identity between isodecoders is shown between parentheses. The rest of the genes with the same anticodon are 100% identical.

^2 ^Underlined anticodons are those that recognize more than one codon; for exemple, the Tyr anticodon GUA recognizes two codons, TAT and TAC, since anticodon Tyr-AUA is missing in trypanosomatids. A1 of the anticodon is modified to inosine (I).

In *T. cruzi*, it was found that three tRNA genes annotated as Val-CAC (Tc00.1047053457717.10, Tc00.1047053483321.10 and Tc00.1047053506321.220) do not seem to correspond to the assigned amino acid (or to any other). They showed only 61% identity with Tc00.1047053506459.249, which we consider is the "real" tRNA-Val-CAC gene, since it is 100% identical to the tRNA-Val-CAC gene from *T. brucei *(and shows 98% identity to its orthologue in *L. major*). Interestingly, we observed that they show 75% identity to the sRNA76 from *T. brucei *(data not shown), which suggests that they might actually encode the orthologue of this gene in *T. cruzi*. Alternatively, they may correspond to tRNAs with undetermined or unknown type. tRNA genes with undetermined type have been found in several species, including *Caenorhabditis elegans*, yeast and human, and their function is unknown. Additionally, Tc00.1047053507579.16, annotated as an Ile-TAT gene, seems to be a pseudogene (or an undetermined tRNA); since it shows only 22% identity to the other two annotated Ile-TAT genes in *T. cruzi *(Tc00.1047053504427.231 and Tc00.1047053508043.11) (data not shown). Also, we identified an extra copy of tRNA-Ala-TGC on contig 8001 (see Additional File 1).

Thus, our analysis indicates the presence of 120 tRNA genes in *T. cruzi*, excluding four genes that might be undetermined tRNAs or encode orthologues of the sRNA76, and including the eight tRNA-Sec genes and the tRNA-Ala-TGC gene (Table 1 and Additional File 1). In *T. brucei *the number of identified tRNA genes is 66, including the two newly identified tRNA-Sec genes and excluding the gene of the sRNA76 orthologue (Fig. 2 and Table 1). In *L. major *there are 83 tRNA genes (Fig. 1 and Table 1), in addition to a pseudogene that we do not include in our analyses. The number of tRNA genes in trypanosomatids is relatively low, considering that eukaryotic organisms usually contain several hundred tRNA genes. For instance, *C. elegans *has 568 tRNA genes, *Homo sapiens *presents 497 tRNA genes and *Saccharomyces cerevisiae *contain 271 tRNA genes [9,10]. In an extreme case, *Danio rerio *(zebra fish) has ~6000 predicted tRNA genes. On the other hand, the microsporidian parasite *Encephalitozoon cuniculi *has only 44 tRNA genes. Bacterial genomes usually have between 29 and 167 tRNA genes in their genomes [9,10].

tRNA genes from eukaryotes typically contain introns, which are usually located between bases 37 and 38 of the anticodon loop. Archaeal tRNA genes also have introns that can be found at the same location of the anticodon loop or in other regions of the tRNA gene. The size of introns is variable, ranging from 7 to 121 bases [9]. In bacterial genomes, a very small number of tRNA-gene introns have been reported, but they correspond to self-splicing introns (group I autocatalytic introns). Analysis of the tRNA genes in Tritryps indicated that only the tRNA-Tyr genes contain an intron; which was previously reported in *T. brucei *[16]. The intron is 11 bases long in *L. major *and *T. brucei*, and 13 bases long in *T. cruzi*; and as in other organisms, it is located between bases 37 and 38 (data not shown). Thus, introns are very rare in Tritryps, not only in protein-coding genes, but also in tRNA genes.

### Isoacceptor tRNA species

Analyses of the anticodon sequences of the tRNA genes in Tritryps showed the presence of 46 isoacceptor types in each of the three species (Table 1) [17]. These 46 isoacceptor types are able to read the 61 codons that specify the canonical amino acids, in addition to Sec, the 21st amino acid. The number of isoacceptor species found in Tritryps is similar to that found in other organisms (from 41 to 55 isoacceptors) [10]. It is important to mention that the two methionine isoacceptors, the initiator and elongator, have been identified (see below), but for practical purposes these two isoacceptors will be considered as one.

Sixteen anticodons were not found in the tRNA genes of trypanosomatids, even though their corresponding codons are present in the protein-coding genes of these organisms [17]. For example, the tRNA with anticodon Ile-GAU is not present in the genome of trypanosomatids, but the codon AUC is present in their protein sequences (Table 1). As mention above, this is possible because some tRNAs are able to recognize more than one codon by allowing flexible base-pairing between the first nucleotide of the anticodon and the third position of the codon (tRNA wobble recognition). Analysis of the data shown in Table 1 indicates that C3 or U3 in the codon are recognized by G1 or A1 of the anticodon (A is converted to the nucleotide inosine in the mature tRNA, which can pair with U3 or C3 in the codon). Thus, trypanosomatids use the A1- or G1-sparing strategy as a decoding mode [9]. This anticodon-choice pattern is similar to that of other eukaryotes such as *C. elegans, H. sapiens *and *A. thaliana*. Other eukaryotic organisms, like yeast and *D. melanogaster*, use the A1- or G1 and C1-sparing strategy [9]. As observed previously [17], the spared anticodons are used equally, since 50% (8/16) of the U3 and C3 codons are read by A1 (or I1) and the remaining 50% are read by G1. In most four-fold degenerate codon families (*i.e. *Leu, Val, Ser, Pro, Thr, Ala and Arg, but not Gly) A1 reads the codons containing U3 and C3, since the corresponding tRNAs with G1 are not present in the Tritryps. We found the same for the Ile family, which contains three codons. On the other hand, all the two-fold degenerate families use G1 to read U3 or C3, given that the tRNAs with A1 are missing. The families that use this strategy are: Asn, Asp, Cys, Gly (although it is fourfold-degenerated), His, Phe, Ser (AGU and AGC codons) and Tyr.

**Figure 3:**
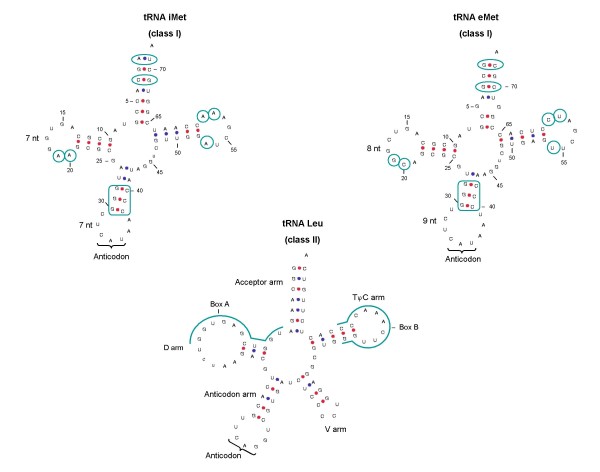
**Secondary structures of initiator and elongator tRNA-Met in *L. major***. Important features for iMet and eMet tRNAs function are indicated. These two molecules correspond to class I tRNAs. The secondary structure of a class II tRNA (Leu) is also presented. The names of the different arms are shown. The position of the internal control elements (Boxes A and B) is also indicated.

The genomes of *L. major, T. brucei *and *T. cruzi *contain four, three and six tRNA-Met genes, respectively [16,17]. Further analysis of these genes indicated that in *L. major *two of them (LmjF09.TRNAMET.01 and LmjF36.TRNAMET.01) correspond to initiator tRNAs (iMet) and two (LmjF11.TRNAMET.01 and LmjF34.TRNA.01) correspond to the elongator form (eMet) (Fig. 3). In *T. brucei*, one iMet (Tb10_tRNA_Met_1) and two eMet genes (Tb04_tRNA_Met_1, Tb04_tRNA_Met_2) were found, although only one of each type was previously reported [16]. Finally, two iMet (Tc00.1047053508231.92, Tc.00.1047053506251.88) and four eMet genes (Tc00.1047053504055.87, Tc00.1047053504055.91, Tc00.1047053506435.327 and Tc00.1047053506435.345) were located in *T. cruzi*. As shown in Fig. 3, the two types of tRNA-Met possess specific features, and most of them were found in the genes from the Tritryps. One of the main characteristics is the highly conserved A:T base pair that is present in all tRNA-iMet in eukaryotes at position A1:U72 (A1:U71 in Tritryps), whereas a G:C pair is found in tRNA-eMet [9]. In yeast, it has been reported that the A1:U72 base pair is the most important determinant for a tRNA-Met to play the role of iMet, since it is necessary for binding to Initiation Factor 2 (eIF2) [18]. When this sequence is mutated, the tRNA-iMet is able to bind to Elongation Factor Tu (EF-Tu) and participates in translation elongation. It is likely that this base pair has a similar function in trypanosomatids.

Other determinants for iMet function are: A53, A58 and A59 in the TψC loop (U54, U59 and C60 in eMet); the base pair C3:G69 (G3:C70 in eMet); bases A19 and A20 (G19 and C20 in eMet); a D-loop composed of 7 nucleotides (8 nt in eMet); and an anticodon loop of 7 nt (9 nt in eMet) [9,19]. All these features are conserved in the Tritryps (Fig. 3). Another distinctive characteristic of the iMet tRNA is the presence of three consecutive G:C pairs at the bottom of the anticodon stem, which is conserved not only in eukaryotes, but also in eubacteria and archaeobacteria. In *E. coli*, it has been shown that mutations in these three consecutive G bases reduce the efficiency of initiation of protein synthesis, by affecting the interactions between the tRNA and the ribosomal P site; thus, these bases are essential to discriminate between initiator and elongator tRNA-Met [9,20]. Tritryps iMet tRNAs have these conserved G:C pairs but, surprisingly, we found them in the eMet tRNAs as well (Fig. 3). Thus, in the Tritryps these base pairs are not a discriminator between iMet and eMet tRNAs, and these organisms must use other features of the iMet to direct it to the P site of the ribosome.

**Figure 4:**
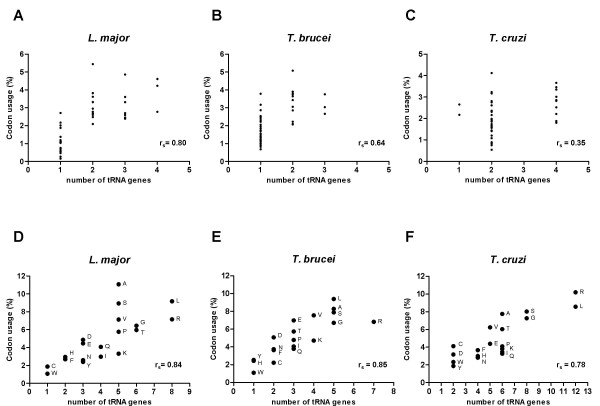
**Correlation between number of tRNA genes and codon usage in Tritryps**. Panels A-C show the correspondence between codon usage and the number of tRNA genes for each isoacceptor (45 types), for the three species (see Table 1). Panels D-F show the correlation between codon usage and the number of tRNA genes per amino acid. Correlation coefficients were evaluated by performing a Spearman test. r_*s *_values are indicated in each panel; p < 0.0001 for panels A, B, D and E; p < 0.02 for panel C; p < 0.0001 for panel F.

In several organisms it has been observed that there is a correlation between tRNA gene copy number and codon usage [21,22]. Apparently, selection on synonymous codon positions causes co-adaptation of codon usage and tRNA content, in order to optimize the effectiveness of protein synthesis [23]. In Tritryps, it has been reported that bias in codon usage correlates with tRNA gene copy number and with protein expression level [17]. This conclusion was made after analyzing around 60,000 codons from highly expressed (tandem duplicated) protein-coding genes from the three parasites. We conducted a similar analysis, but including all the 8272 protein coding genes from *L. major *(5,249,748 codons), and 5119 randomly selected genes from *T. brucei *(2,620,035 codons), as well as 1779 genes from *T. cruzi *(986,435 codons). We plotted codon usage (see Table 1) against the number of tRNA genes for each isoacceptor, for the three species, and a possible correlation was evaluated by the Spearman test (Figure 4, panels A-C). The data indicated a positive correlation between these variables for *L. major *(r_*s *_= 0.80) and *T. brucei *(r_*s *_= 0.64), which indicates that, similar to other organisms, codon usage patterns seem to be co-adapted with the relative abundance of the corresponding tRNAs in these parasites. However, in the case of *T. cruzi*, the obtained Spearman value (r_*s *_= 0.35) indicated a low degree of correlation between the two variables. This may reflect the fact that the *T. cruzi *strain used for the sequencing project is a hybrid of two strains, and some of their genes might be duplicated, while others might not be; as shown in Fig. 4C and Table 1, in *T. cruzi *the vast majority of the isoacceptor species are encoded by either two or four genes (only two isoaceptors have one gene, and none of them has three genes), whereas in *L. major *and *T. brucei *a high number of the isoacceptors are encoded by a single gene. The correlation analysis was repeated, but now plotting the percentage of codon usage *versus *the number of tRNA genes per amino acid (Fig. 4, panels D-F). This time, the Spearman value was high in *T. cruzi *(r_*s *_= 0.78), indicating a strong correlation between both parameters. As before, strong correlations were found in *L. major *(r_*s *_= 0.84) and *T. brucei *(r_*s *_= 0.85).

### Organization of tRNA genes

In *L. major*, the 83 tRNA genes are distributed among 31 loci, on 19 different chromosomes (Fig. 1 and Additional File 1). Most tRNA genes are organized into clusters of 2 to 10 genes, on either top or bottom strand, which may contain other Pol III-transcribed genes. For example, in the locus located on chromosome 23 (chr23 in Fig. 1) there are 10 tRNA genes, a 5S rRNA gene and the U1 and U3 snRNA genes. Locus IV on chromosome 36 (chr36-IV) has four tRNA genes and the U5 snRNA gene. The eleven 5S rRNA genes found in the *L. major *genome are distributed in six chromosomes, and are always associated to tRNA genes (Fig. 1 and Additional File 1). Only eight loci contain single tRNA genes (chr03, chr06, chr07, chr16, chr24-I, chr24-III, chr30 and chr36-III). In most cases, intergenic regions that separate Pol III-transcribed genes are short, with an average size of 202 bases (Fig. 1). However, they can be as small as 35 bases (intergenic region between tRNA-eMet and tRNA-Leu on cluster chr34-I) and as long as 5406 bases (intergenic region between tRNA-Gly and tRNA-Ala on cluster chr11-I). Intergenic regions between the protein-coding genes that flank the clusters and the first or last Pol III gene of the cluster are normally longer than the ones that separate Pol III genes. The average length of such regions is 1490 bp, with a minimum of 238 bp (intergenic region between tRNA-Lys and the "right" Pol II gene on cluster chr21-II) and a maximum of 7949 pb (intergenic region between tRNA-Glu and the "left" Pol II gene on cluster chr09-III) (Fig. 1). The mean length of intergenic regions between protein-coding genes in the *L. major *genome is 2045 bp [1].

In *T. brucei*, the 66 tRNA genes are located on 26 loci, on eight different chromosomes (Fig. 2 and Additional File 1). As in *L. major*, in *T. brucei *the number of tRNA genes per cluster ranges from 2 to 10. Eleven of the 66 tRNA genes are single genes in *T. brucei *(loci chr04-I, chr05-I, chr05-II, chr07-I, chr07-III, chr07-IV, chr08-IV, chr09-I, chr09-II, chr09-III and chr11-I). Similarly to *L. major*, intergenic regions that separate Pol III genes in *T. brucei *are short in most cases (average length is 327 bp, ranging from 43 to 3172 bp) (Fig. 2). Regarding intergenic regions between the protein-coding genes that flank the clusters and the first or last Pol III gene of the cluster, the mean size is 2473 bp (showing a range from 34 to 10415 bp). In the *T. brucei *genome the average length of intergenic regions between protein-coding genes is 1279 bp [2]. Since it has not been possible to assemble fully adjacent sequences for the chromosomes of *T. cruzi*, at the present we are unable to determine the genomic organization of the tRNA genes in this parasite. In contrast to *L. major*, the 5S rRNA genes in *T. brucei *and *T. cruzi *are organized into tandem arrays, which are not associated to tRNA genes [24,25].

In most eukaryotic organisms, tRNA genes seem to be dispersed randomly throughout the genome. However, in human cells the distribution is non-random, since more than 25% of the tRNA genes are located in a region of only about 4 Mb on chromosome 6. This region represents only 0.1% of the human genome, but contains an almost complete set of tRNA genes. Moreover, 280 out of 497 tRNA genes (more than half) are found on either chromosome 1 or chromosome 6 [26]. The distribution of genes in the *L. major *genome does not seem to be totally random, since half of the chromosomes do not contain even a single tRNA gene. Additionally, 60 tRNA genes (72%) are located in only 7 chromosomes (9, 11, 23, 24, 31, 34 and 36), which represent only 26% of the genome (Fig. 1 and Additional File 1). In *T. brucei*, 40 (61.5%) of the tRNA genes are found in just 3 chromosomes (4, 7 and 8), which is only about 24% of the genome (Fig. 2 and Additional File 1). tRNA genes in *S. cerevisiae*, though dispersed in the linear genome, co-localize with 5S rDNA at the nucleolus. Nucleolar localization requires tRNA gene transcription, because inactivation of the internal promoter eliminates its nucleolar location [27]. It remains to be tested whether tRNA genes in trypanosomatids show such a specific cellular localization.

In *Schizosaccharomyces pombe *and *C. elegans*, tRNA genes are often clustered in centromeres [28]. These tRNA genes contribute to centromere function by defining domain boundaries important for centromere activity [29]. Putative centromeric regions have been reported in a few chromosomes in *T. cruzi *and *T. brucei *[30], where they localize to strand-switch regions that separate divergent PGCs. While these regions do not seem to contain tRNA genes, two clusters of tRNA genes in *L. major *(chr09-II and chr10), and one cluster in *T. brucei *(chr04-I) are located in divergent strand-switch regions (Figs. 1 and 2), and thus might be candidates to contain centromeric regions. Therefore, it is possible that in trypanosomatids, like *S. pombe *and *C. elegans*, some tRNA genes might be important for centromeric activity.

### Spatial relation between Pol III and Pol II genes

We have previously shown that transcription of two convergent PGCs on *L. major *chromosome 3 terminates on the convergent strand-switch area, within the tRNA-gene region [6]. Interestingly, 14 of the 39 convergent strand-switch regions (35.9%) in the *L. major *genome contain at least one tRNA gene (Fig. 1), representing 45.2% of the 31 tRNA loci. A similar situation was found in *T. brucei*, where 34.6% of the tRNA loci are located within convergent strand-switch regions (Fig. 2). This suggests that the use of tRNA genes as signals for termination of transcription of convergent clusters of protein-coding genes might be a common process in trypanosomatids. Indeed, recent evidence suggest that this is the case for tRNA clusters located within PGCs, since peaks of acetylated histone H3 are found immediately downstream of the tRNA cluster in all cases [31]. Acetylated histones are markers for open chromatin in all eukaryotes and have been found at the 5' end of all polycistronic gene clusters in *L. major*.

### Synteny of Pol III genes

**Figure 5:**
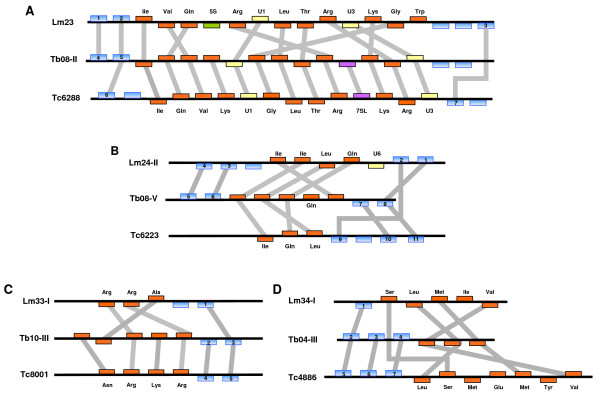
**Comparative order of Pol III-transcribed genes in Tritryps**. Clusters that present some degree of synteny among Tritryps are shown. In *L. major*, they correspond to loci chr23 (panel A), chr24-II (panel B), chr33-I (panel C) and chr34-I (panel D). The order of genes in clusters chr24-II and chr34-I was inverted compared to the maps shown in figure 1. The corresponding orthologous regions in *T. brucei *and *T. cruzi *are indicated. Orthologous genes are joined by grey lines. Figure is not to scale.

It has been found that the genomes of the Tritryps are highly syntenic, that is to say, they show conservation of gene order, with the *T. brucei *and *L. major *genomes containing 110 blocks of synteny spanning 19.9 and 30.7 Mb, respectively [7]. Many of these synteny blocks correspond to intact PGCs, which are transcribed by Pol II. In contrast, the majority of the tRNA clusters do not show synteny, but a few of them do show conservation (Fig. 5). Among the latter, the most remarkable example is a cluster of 13 Pol III genes that is highly syntenic; corresponding to chr23 in *L. major*, chr08-II from *T. brucei*, and the cluster located on contig Tc6288 from *T. cruzi *(Fig. 5A). Surprisingly, the order of the genes in this cluster is identical between *T. brucei *and *T. cruzi*, although the U1 snRNA, the 7SL RNA and the tRNA-Leu genes are located on different strands. Most of the 13 Pol III-transcribed genes are present in the *L. major *cluster, but their order is not identical to either of the other two clusters (Fig. 5A). Additionally, a 5S rRNA gene replaced a 7SL RNA gene and a tRNA-Trp gene replaced one of the tRNA-Lys genes. Another Pol III-gene cluster that exhibits synteny is chr24-II in *L. major*, chr08-V in *T. brucei *and contig Tc6223 from *T. cruzi *(Fig. 5B). Here, we found tRNA genes for Ile, Leu and Gln that are syntenic among the three species. A second copy of a tRNA-Ile is conserved between *L. major *and *T. brucei*. One difference is that the *L. major *cluster contains an U6 snRNA gene that is replaced by a tRNA-Gln gene in *T. brucei*. Other syntenic tRNA clusters are chr33-I from *L. major*, chr10-III from *T. brucei *and contig Tc8001 from *T. cruzi *(Fig. 5C), and chr34-I from *L. major*, chr04-III from *T. brucei *and contig Tc4886 from *T. cruzi *(Fig. 5D). Several of the protein-coding genes that flank these four syntenic tRNA-gene clusters are also syntenic among Tritryps (Fig. 5). As in Tritryps, an overall weak synteny of Pol III-transcribed genes has been observed between two species of the oomycete *Phytophthora *[32], indicating a reduced importance of genome location of Pol III genes compared to protein-coding genes.

### Consensus sequences of promoter elements

**Figure 6:**
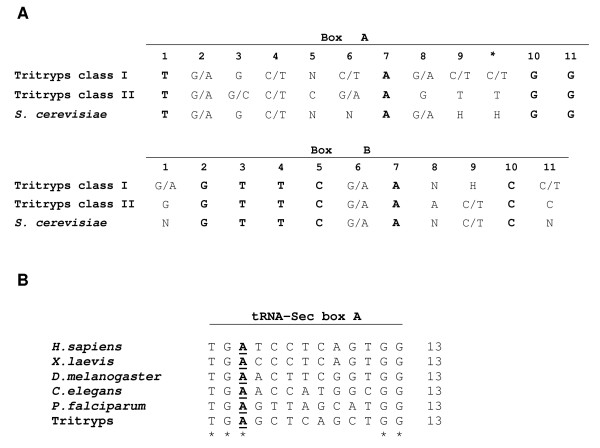
**Consensus sequences for Box A and Box B from tRNA genes in Tritryps**. Sequences from class I and class II tRNA genes from Tritryps are compared to the *S. cerevisiae *consensus sequences (panel A). In Box A, some tRNA genes contain an extra base between positions 9 and 10 (marked with an asterisk) (see text). Conserved positions are shown in bold type. Panel B shows a comparison of Box A from selenocysteine tRNA genes from the indicated species. Conserved nucleotides are indicated with an asterisk. An A in the third position (in bold and underlined) seems to be specific to Box A from Sec genes. H represents C, T or A.

One of the distinctive features of most genes transcribed by Pol III is that their promoter sequences are internal, located within the transcribed region. In the case of tRNA genes, the promoter consists of two conserved elements: Boxes A and B [33]. While Box A is normally positioned close to the transcription start site, the location of Box B is variable, partly because some tRNAs have short introns within the coding region. In contrast, tRNA genes in prokaryotic cells contain promoter elements similar to those found in protein-coding genes: the start-point (usually a purine), the -10 sequence (the TATA Box) and the -35 sequence (the hexamer) [34]. Consensus sequences of trypanosomatid tRNA promoter elements (Fig. 6A) were determined by analyzing the sequences of all tRNA genes in *L. major*, *T. brucei *and *T. cruzi *and comparing them to the sequences of Boxes A and B from *S. cerevisiae *[9]. The tRNAs were divided into two classes, depending on the size of the variable loop (Fig. 3). Class I tRNAs have a short variable loop of 4 or 5 nucleotides, whereas class II tRNAs posses a long variable arm, with a double helical stem of 3 to 7 base pairs and a loop of 3 to 5 nucleotides (Fig. 3) [9]. In the Tritryps, 43 genes belong to class II (all Leu and Ser tRNA genes, but excluding tRNA-Sec genes), and 215 genes are class I. Since we observed sequence differences between class I and class II tRNA genes, we analyzed them separately. Half of the bases from the consensus sequence of Box A (positions 1, 2, 4, 7, 10 and 11) are identical between class I and class II genes (and identical to the *S. cerevisiae *consensus sequence) (Fig. 6A). However, position 5 is different between both classes, since class I genes may have any nucleotide, whereas class II genes always have a C. Also, class I tRNA genes present C or T at position 6, while class II genes always have A or G. Regarding Box B, position 8 is different between both classes: an A is always present in class II genes, while class I genes may have any nucleotide. Around 20% of the class I tRNA genes in Tritryps have an additional nucleotide in Box A, between positions 9 and 10 (marked with an asterisk in Fig. 6A). These tRNA genes are: eMet-CAT, Asn-GTT, Ile-AAT, Ile-TAT, Lys-CTT, Phe-GAG and Tyr-GTA. Regarding class II tRNAs, the Leu-TAA genes have a T between positions 9 and 10 of Box A (Fig. 6A).

A few exceptions to the consensus sequence were found among Tritryps. For Box A from class I genes, these include the following: four tRNA-Val genes (LmjF09.VAL.01, LmjF09.VAL.02, Tb08_tRNA_Val_1 and Tc00.1047053506459.249) have a C at position 2 (instead of A or G); all four tRNA-Val-TAC genes (LmjF23.VAL.01, Tb08_tRNA_Val_1, Tc00.1047053504427.233 and Tc00.1047053508043.13) present an A at position 3 (instead of G); all six tRNA-Ala-AGC genes (LmjF17.TRNAALA.01, LmjF31.TRNAALA.01, Tb07_tRNA_Ala_1, Tb07_tRNA_Ala_2, Tc00.1047053510057.40 and Tc00.1047053508909.130) have an extra base (an A) between positions 8 and 9; and the six tRNA-Val-AAC genes from Tritryps (LmjF21.TRNAVAL.01, LmjF34.TRNAVAL.01, Tb_04_tRNA_Val_1, Tb_04_tRNA_Val_2, Tc00.1047053506435.363 and Tc00.1047053504055.95) also have an additional base (a G) between positions 8 and 9.

Concerning class II genes, the exceptions to Box A consensus sequence are the four tRNA-Leu-TAA genes present in the Tritryps genomes (LmjF24.TRNALEU.01, Tb08_tRNA_Leu_2, Tc00.1047053510721.13 and Tc00.1047053511909.9), which have a T at position 2 (instead of G or A). In regard to Box B, the genes that do not have the consensus sequence are: LmjF09.TRNAHIS.01 and LmjF09.TRNAHIS.02 present a C at position 4; Tb07_tRNA_Ala_3 has a T at position 5; Tc00.1047053511241.10 presents an A at position 10; and tRNA-Ala-AGC and tRNA-iMet genes have an A at position 3 (data not shown).

Analysis of the promoter sequences from tRNA-Sec genes in Tritryps indicated that Box A contains an additional A between bases 2 and 3, compared with the consensus sequences (see Fig. 6B). This insertion was previously reported in tRNA-Sec genes from other organisms [35] (Fig. 6B). Regarding Box B, tRNA-Sec genes from Tritryps present two changes compared to the highly conserved consensus sequence: a C at position 1 (instead of a G) and a G at position 11 (in lieu of C) (data not shown). In other species, the sequence of Box B from tRNA-Sec is identical to the corresponding consensus sequence. In *Xenopus laevis*, transcription of tRNA-Sec genes is directed by three extragenic domains (a TATA Box, a proximal sequence element and an activator element) and Box B. Apparently, Box A is not part of the promoter [36,37]. Since both internal control elements from Sec genes in Tritryps differ from the corresponding consensus sequences, it is possible that synthesis of tRNA-Sec is regulated only by external elements in these parasites. We are currently exploring this possibility.

### tRNA isodecoder genes

**Figure 7:**
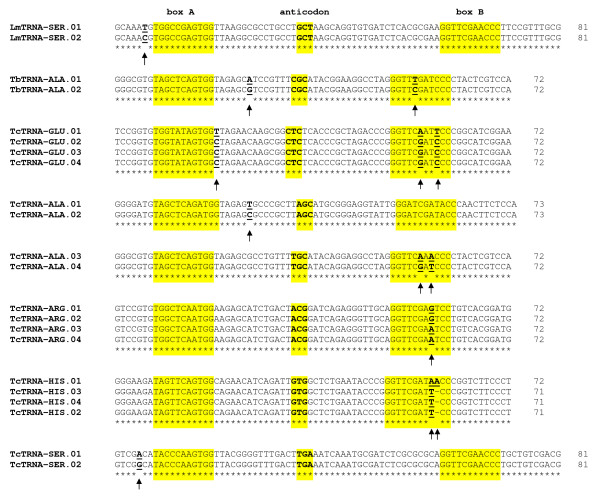
**Sequence comparison of tRNA Isodecoder genes in the Tritryps**. One isodecoder tRNA gene was found in *L. major *(LmTRNA-Ser) and *T. brucei *(TbTRNA-Ala), and six were found in *T. cruzi *(TcTRNA-Glu, -Ala.01, -Ala.03, -Arg, -His and -Ser). Bases that show variation are indicated in bold and underlined, and marked with an arrow. The position of internal control elements (Boxes A and B) and the anticodon are indicated. The genes included in this figure are the following (GeneDB names in parentheses): LmTRNA-SER.01 (LmjF17.TRNASER.01), LmTRNA-SER.02 (LmjF21.TRNASER.01), TbTRNA-ALA.01 (Tb07_tRNA_Ala_3), TbTRNA-ALA.02 (Tb11_tRNA_Ala_1), TcTRNA-GLU.01 (Tc00.1047053506435.336), TcTRNA-GLU.02 (Tc00.1047053504055.89), TcTRNA-GLU.03 (Tc00.1047053508999.180), TcTRNA-GLU.04 (Tc00.1047053510959.8), TcTRNA-ALA.01 (Tc00.1047053510057.40), TcTRNA-ALA.02 (Tc00.1047053508909.130), TcTRNA-ALA.03 (Tc00.1047053475029.40), TcTRNA-ALA.04 (gene located in contig 8001, not annotated in geneDB), TcTRNA-ARG.01 (Tc00.1047053504427.243), TcTRNA-ARG.02 (Tc00.1047053506619.59), TcTRNA-ARG.03 (Tc00.1047053508043.23), TcTRNA-ARG.04 (Tc00.1047053511191.29), TcTRNA-HIS.01 (Tc00.1047053511241.10), TcTRNA-HIS.03 (Tc00.1047053508087.5), TcTRNA-HIS.04 (Tc00.1047053508861.10), TcTRNA-HIS.02 (Tc00.1047053506663.10), TcTRNA-SER.01 (Tc00.1047053510057.50), TcTRNA-SER.02 (Tc00.1047053508909.120).

Sequence analysis of isoacceptor tRNAs in several organisms indicated the presence of tRNA isodecoder genes (tRNA genes having the same anticodon but different sequences elsewhere in the tRNA body) [10]. In eukaryotes, the number of isodecoder genes ranges from 10 (yeast) to 246 (chimp), while in bacterial genomes the number of isodecoders varies from 0 to 26 [10]. By comparing the sequences of isoacceptor tRNAs in the Tritryps, one isodecoder gene was found in *L. major *(tRNA-Ser-GCT), and one was found in *T. brucei *(tRNA-Ala-CGC) (Fig. 7 and Table 1). Since there are only two copies of the corresponding isoaceptor class in each case, we have arbitrarily designated LmtRNA-Ser.01 (LmjF17.TRNASER.01) and TbtRNA-Ala.01 (Tb07_tRNA_Ala_3) as the isodecoders. Sequence identity between the isodecoder and the "majority member" is 98% in *L. major *and 97% in *T. brucei*. While in *L. major *the sequence difference locates near Box A, in *T. brucei *one of the two observed differences lies in one of the conserved bases of Box B (Fig. 7). In *T. cruzi*, six isodecoder genes were identified: TctRNA-Glu.01 (Tc00.1047053506435.336), TctRNA-Ala.01 (Tc00.1047053510057.40), TctRNA-Ala.03 (Tc00.1047053475029.40), TctRNA-Arg.01 (Tc00.1047053504427.243), TctRNA-His.01 (Tc00.1047053511241.10) and TctRNA-Ser.01 (Tc00.1047053510057.50) (Table 1 and Fig. 7). In four cases (TctRNA-Glu.01, TctRNA-Ala.03, TctRNA-Arg.01 and TctRNA-His.01), sequence differences were located to variable nucleotides from Box B (Fig. 7). As in Tritryps, sequence variations between human tRNA isodecoders have been located within internal control elements [10]. In such cases, changes were found in variable nucleotides from Boxes A and B. Thus, the occurrence of changes within internal control elements in tRNA isodecoder genes suggests that differential regulation of Pol III transcription is possible in Tritryps; the fact that the highly conserved C at position 10 of Box B from TbtRNA-Ala.02 is changed to T in the corresponding tRNA isodecoder (TbtRNA-Ala.01) (Fig. 7) strongly supports this possibility. Sequence changes in isodecoders are not only restricted to internal control elements, but they might be present all along the tRNA body (Fig. 7) [10]. Therefore, the diversity of tRNA genes is much higher than originally thought. The functional meaning of such diversity has yet to be investigated.

### Signals for transcription termination

**Figure 8:**
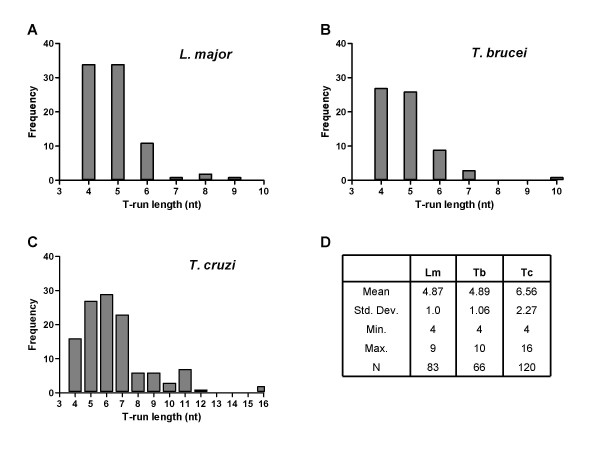
**Length distribution of termination signals from tRNA genes in Tritryps**. The size of the run of Ts in every tRNA gene from *L. major *(panel A), *T. brucei *(panel B) and *T. cruzi *(panel C) was plotted against frequency. Statistical data is shown in panel D.

A cluster of several T residues in the coding DNA strand acts as a signal to terminate Pol III transcription [33]. The cluster of Ts is usually located within the first 30 bases following the gene. In human and mice, Pol III needs four Ts to end transcription, and tRNA genes that have five or more Ts are very rare in these species. On the other hand, in the genomes of *S. pombe *and *S. cerevisiae *the majority of the tRNA genes have six and seven Ts, respectively [38,39]. Interestingly, they do not have any single gene whose termination signal is shorter than five Ts. For a particular species, termination efficiency tends to increase with the length of the T run. In *L. major*, it has been shown that transcription of the tRNA located on chromosome 3 terminates within a tract of four Ts [6]. To gain insight into Pol III termination signals in trypanosomatids, we decided to analyze the sequences downstream of all the tRNA genes. A cluster of Ts of variable length was found on every single tRNA gene in the Tritryps (see Additional File 2); the distance between the end of the gene and the run of Ts varies from zero to seven bases. In *L. major*, the mean length of the run of Ts is 4.87 bases, with a minimum of four and a maximum of nine Ts (Fig. 8, panels A and D). Similar results were obtained in *T. brucei*, where the average T-run length is 4.89 bases (ranging from four to ten Ts) (Fig. 8, panels B and D). In the tRNA genes from *T. cruzi*, however, the stretches of Ts are longer, showing a mean length of 6.56, with two genes presenting a run of 16 consecutive T residues (Fig. 8C and 8D).

The presence of a second stretch of Ts that acts as a potential "back up" termination signal is a common feature in tRNA genes from eukaryotes [38]. The second run of Ts is normally located within the first 30 bp downstream of the first one. In the case of *S. cerevisiae*, *S. pombe*, *H. sapiens *and *Mus musculus*, the percentages of tRNA genes that have a back up T-run are 44, 53, 31 and 33%, respectively [38]. Analysis of the sequences downstream of the T-runs in *T. cruzi *showed that 58 tRNA genes (48.3%) have a back up T-run, whose length is between 4 to15 bases (Additional File 2). Therefore, the percentage of tRNA genes with a second run of Ts in *T. cruzi *is very similar to that found in *S. cerevisiae *and *S. pombe*. Interestingly, only 13 tRNA genes in *L. major *(15.6%) and 18 genes in *T. brucei *(27%) present a back up T-track (which is from 4 to 10 bases long) (see Additional File 2); these percentages are even smaller than those found in mammals. Thus, in *L. major *and *T. brucei *a single and short run of Ts seems to be sufficient to achieve proper transcription termination in the majority of the tRNA genes. On the other hand, *T. cruzi *seems to require longer T-runs, in addition to a second T stretch, to direct transcription termination. This indicates that the mechanism of Pol III transcription termination in *L. major *and *T. brucei *might be different from that one in *T. cruzi *and other eukaryotes. Sequences downstream and upstream of the run of Ts might contribute to the strength of the terminator, as observed in some tRNAs in *S. cerevisiae *[38].

Although most tRNA genes are clustered in trypanosomatids, the presence of runs of Ts located downstream of all the tRNA genes suggests that they are transcribed as monocistrons, which is common among eukaryotes. In prokaryotic cells genes encoding tRNAs are transcribed in either a monocistronic or polycistronic manner. In the latter case, an RNA containing several tRNA precursors in tandem is processed to yield functional tRNAs [40]. In plants, dicistronic transcripts containing a tRNA and a snoRNA have been found [41]. Moreover, the presence of precursor RNA molecules that contain both a tRNA and a mRNA has been reported in *E. coli *[42].

## Conclusion

In comparison to most eukaryotic organisms, Tritryps present a low number of tRNA genes. A total of 46 isoacceptor types were identified, which are able to read the 61 codons that specify the canonical amino acids, in addition to Sec. Trypanosomatids use the A1- or G1-sparing strategy as a decoding mode, by allowing flexible base pairing between G1 or A1 of the anticodon and C3 or U3 in the codon. Most tRNA genes in Tritryps are organized into clusters (from 2–10 genes) that may contain other Pol III genes. Some of the clusters show a remarkable conservation of gene order among Tritryps. The distribution of tRNA genes in the genomes of *L. major *and *T. brucei *does not seem to be totally random. We also found that 14 of the 39 convergent strand-switch regions present in the *L. major *genome are separated by at least one tRNA gene, which raise the possibility that other tRNA genes (in addition to the one present on chromosome 3) are involved in termination of Pol II transcription of convergent PGCs in this parasite. A run of Ts of variable length was found downstream of all the 269 tRNA genes present in the Tritryps. In *T. cruzi *the clusters of Ts are larger than in *L. major *and *T. brucei *(an average of 6 Ts *versus *5 Ts, respectively); moreover, the presence of a back up T run is more common in *T. cruzi *than in the other two Tritryps. Analysis of the internal promoter elements allowed us to establish consensus sequences for Boxes A and B of class I and class II tRNA genes. Interestingly, special characteristics were found in Boxes A and B from tRNA-Sec genes in Tritryps, which suggests that the mechanisms that regulate their transcription might be different from those of other tRNA genes. Lastly, we have identified several tRNA isodecoder genes in the Tritryps, especially in *T. cruzi*. The fact that in some cases the sequence differences occur within the internal promoter elements suggests the possibility of differential expression of tRNA genes in these organisms.

## Methods

All tRNA genes annotated in the *L. major*, *T. brucei *and *T. cruzi *genome databases  (versions 2.1) were analyzed with the tRNAscan-SE program [43] to verify the presence and identity of the tRNAs. In addition to the examination of all the features associated to the typical tRNA cloverleaf structure, we also analyzed the presence of internal promoter elements (Boxes A and B) and T-tracts at the 3' end of the tRNA gene (which should be present in tRNA genes, but not necessarily in pseudogenes). Sequence comparisons among putative tRNA isoacceptors were performed using the ClustalW2 program . Information for the genomic and synteny maps was obtained from the GeneDB databases. BLAST searches were performed in these databases to locate the tRNA-Sec genes [13], as well as the sRNA76 [12]. Codon usage data was obtained from the Sanger site  for *L. major*, and from the Kazusa web page  for *T. brucei*. Codon usage data for *T. cruzi *was calculated by analyzing coding sequences (obtained from the Sanger site) in the codon usage page from SMS . The Spearman correlation analysis and the descriptive statistical analysis of T-run data were performed with the GraphPad Prism5 program .

## Authors' contributions

NPM performed data collection, synteny analysis, sequence comparisons, location of tRNA-Sec genes and the statistical analysis of the data. She also carried out the analysis of the transcription terminations signals and helped draft the manuscript. LEFM analyzed the tRNA genes with the tRNAscan-SE program and helped draw the genome maps. EEFA helped collect data from the *T. brucei *databases, and analyzed the sequences to obtain the consensus sequences for boxes A and B. RGMC was responsible for data collection from the *T. cruzi *database and participated in the codon usage analysis. RHR helped draft the manuscript and participated in the statistical analysis of the data. PJM participated in the writing and editing of the manuscript. SMC conceived the study, analyzed data and wrote the manuscript. All authors read and approved the manuscript.

## Supplementary Material

Additional file 1 **Table S1. List of tRNA genes in the Tritryps**. In *L. major *and *T. brucei*, genes are shown by chromosomal location. Other Pol III-transcribed genes that are associated to tRNA genes are also listed. In *T. brucei*, the names of the genes correspond to the temporary systematic name. For those genes that already have a permanent systematic name in the GeneDB database, it is indicated between parentheses. tRNA genes in *T. cruzi *are organized by amino acid. Click here for file

Additional file 2 **Table S2. tRNA-gene transcription termination signals in Tritryps**. The sequences shown start immediately downstream of the end of the tRNA genes. Clusters of Ts are shown in bold type. Those genes that present a second (back up) T-run of 4 or more bases are highlighted in yellow. Click here for file
